# Genome-wide identification, evolution and expression analysis of the aspartic protease gene family during rapid growth of moso bamboo (*Phyllostachys edulis*) shoots

**DOI:** 10.1186/s12864-020-07290-7

**Published:** 2021-01-10

**Authors:** Xiaqin Wang, Xinyang Yan, Shubin Li, Yun Jing, Lianfeng Gu, Shuangquan Zou, Jin Zhang, Bobin Liu

**Affiliations:** 1 College of Forestry, Fujian Agriculture and Forestry University, Fuzhou, 350002 China; 2 Fujian Colleges and Universities Engineering Research Institute of Conservation & Utilization of Natural Bioresources, Fujian Agriculture and Forestry University, Fuzhou, 350002 China; 3 State Key Laboratory of Subtropical Silviculture, School of Forestry and Biotechnology, Zhejiang A&F University, Zhejiang, 311300 Hangzhou China

**Keywords:** Aspartic protease, Moso bamboo, Programmed cell death, Rapid growth

## Abstract

**Background:**

Aspartic proteases (APs) are a class of aspartic peptidases belonging to nine proteolytic enzyme families whose members are widely distributed in biological organisms. APs play essential functions during plant development and environmental adaptation. However, there are few reports about APs in fast-growing moso bamboo.

**Result:**

In this study, we identified a total of 129 AP proteins (*PhAP*s) encoded by the moso bamboo genome. Phylogenetic and gene structure analyses showed that these 129 *PhAP*s could be divided into three categories (categories A, B and C). The *PhAP* gene family in moso bamboo may have undergone gene expansion, especially the members of categories A and B, although homologs of some members in category C have been lost. The chromosomal location of *PhAP*s suggested that segmental and tandem duplication events were critical for *PhAP* gene expansion. Promoter analysis revealed that *PhAP*s in moso bamboo may be involved in plant development and responses to environmental stress. Furthermore, *PhAP*s showed tissue-specific expression patterns and may play important roles in rapid growth, including programmed cell death, cell division and elongation, by integrating environmental signals such as light and gibberellin signals.

**Conclusion:**

Comprehensive analysis of the AP gene family in moso bamboo suggests that *PhAP*s have experienced gene expansion that is distinct from that in rice and may play an important role in moso bamboo organ development and rapid growth. Our results provide a direction and lay a foundation for further analysis of plant AP genes to clarify their function during rapid growth.

**Supplementary Information:**

The online version contains supplementary material available at 10.1186/s12864-020-07290-7.

## Background

Aspartic proteinases (APs; Enzyme Commission 3.4.23) are proteolytic enzymes and play important roles in protein maturation and degradation [1, 2]. The majority of APs have two conserved motifs with catalytic activity: an Asp-Thr-Gly (DTG) motif and an Asp-Ser-Gly (DSG) motif [3, 4]. APs are widely distributed among microbes, mammals and plants [3, 5] and are divided into 16 subfamilies based on their tertiary structure and evolutionary relationships [4, 6]. APs are involved in many important biological processes that are involved in development, nutrition, pathogenesis, disease and so on and have potential for commercial application [7, 8].

Most plant APs are grouped into the A1 family and exhibit the two basic features of A1 family members: one features is that they are active under acidic conditions, and the other is that their catalytic activity can be specifically inhibited by pepstatin A [1, 9]. Since the 1980s, plant APs have been purified via pepstatin A-agarose columns and detected in various plant species [3, 4, 10]. Plant APs can be classified into three categories: typical APs, nucellin-like APs and atypical APs [1, 9]. Typical APs contain a plant-specific insert (PSI) similar to that of saposin-like proteins, but it is removed during protein maturation [1, 2]. Nucellin-like APs are similar to nucellins in barley ovules [11]. The characteristics of atypical APs are intermediate between those of typical and nucellin-like APs [9, 12]. Pepstatin A activity has been detected in immature, mature, and germinated seeds in wheat, and the expression pattern showed a role of APs in regulating protein degradation [13, 14]. Plant APs are also considered to be responsible for protein processing and degradation, such as plant senescence, programmed cell death (PCD), reproduction, and stress responses [2, 15–20], which are critical for plant development. With the development of DNA sequencing technology, members of plant AP gene families have been identified in *Arabidopsis* [9], rice [12], grape [21], and poplar [22], revealing gene expansion and functional diversity [12, 22].

The function of plant APs has been determined primarily in seeds, including dormant seeds and different parts of seeds [10, 14, 23, 24]. It was proposed that, during seed development, plant APs are involved in seed storage protein processing on the basis of the 2S albumin process in vitro and colocalization with proteins in the plant body [25]. During seed germination, plant APs are considered to be involved in seed storage protein degradation [26–28]. Recently, *Arabidopsis ASPARTIC PROTEASE IN GUARD CELL 1* (*ASPG1*) was reported to promote seed germination by accelerating the breakdown of seed storage proteins [28]. In addition to their involvement in seed development and germination, APs participate in the degradation of insect proteins, allowing carnivorous plants to obtain nitrogen from those sources [15, 29]. Plant APs also play roles in the response to biotic and abiotic stresses. *ASPG1* is abscisic acid (ABA) inducible, and *Arabidopsis* plants overexpressing this gene had in increased ability to resist drought stress because of the participation of the transgene in ABA-dependent responsiveness [17]. *Constitutive Disease Resistance 1* (*CDR1*), an atypical plant aspartic proteinase, exhibits the ability to induce systemic defense responses against bacterial and fungal pathogens in rice and *Arabidopsis* [20, 30, 31]. Ectopic expression of *VqAP13*, a grape aspartic protease gene, can afford powdery mildew resistance but reduces *Botrytis cinerea* resistance by regulating the salicylic acid and MeJA signaling pathways [19]. Plant APs also play roles in plant development, such as reproduction and lateral root formation. *OsAP65* has been proposed to be involved in biosynthesis of compounds that are essential to pollen germination and pollen tube growth in rice [32]. Two novel *AtAP*s in *Arabidopsis* (*A36* and *A39*) have been speculated to participate in gametogenesis and pollen guidance [18]. Recently, an atypical aspartic protease, *Atypical Aspartic Protease in Roots 1* (*ASPR1*), was determined to suppress primary root growth and lateral root development [33]. Altogether, plant APs are important proteins that are involved in various aspects of plant development and responses to environmental changes.

Some plant APs also play an important role in regulating PCD. In barley, a gene encoding an aspartic protease-like protein (‘nucellin’) was highly expressed after pollination, which was synchronized to nuclear cell degeneration characteristic of PCD [11]. Phytepsin, a vacuolar aspartic proteinase that is a plant homolog of cathepsin D and mediates PCD in barley, is highly expressed during the active autolysis of the root cap and in tracheary elements and sieve cells [34]. In rice, the transcripts of *OsAP25* and *OsAP37* in anthers are activated by *ETERNAL TAPETUM 1* (*EAT1*) to regulate PCD in tapetal cells [35]. In *Arabidopsis*, *PROMOTION OF CELL SURVIVAL 1* (*PCS1*) encodes an aspartic protease, and compared with wild type, loss-of-function mutants experience gametophyte degeneration and cell death of developing embryos [36]. AP proteins have also been identified in the plant cell wall, and *cis*-elements related to secondary cell wall (SCW) thickening and PCD, such as SNBE, TERE, and SMRE, were discovered upstream of partial AP genes from poplar, strongly suggesting that APs play important roles in SCW and PCD [37–41]. To date, there are many reports on plant AP function in model plant species such as *Arabidopsis* and rice. However, the function of APs in rapid-growing plant species such as bamboo is still unclear.

Bamboo is a member of the *Gramineae* family, is widely distributed worldwide and is a rapid-growing plant species. Bamboo forests can provide young bamboo shoots for food, fibrous raw material, building materials, raw materials for furniture and crafts and so on within a short time [42]. In addition to its economic benefits, bamboo also has important ecological functions, such as the ability to restore degraded landscapes and combat global climate change [42, 43]. The moso bamboo (*Phyllostachys edulis*) planting area is approximately 3.27 million ha and constitutes most of the bamboo forest region in China [43]. Rapid growth of moso bamboo occurs after the young bamboo shoots are covered with a shell and emerge from the ground. PCD was revealed to occur in pith cavity formation during rapid bamboo growth [44]. During the bamboo rapid-growth stage, cell division gradually decreases, while cell elongation and secondary cell wall thickening also occur [45, 46]. Therefore, PCD and SCW formation are important biological events during rapid growth of moso bamboo. Members of the NAC, MYB and LAC gene families have been identified as being associated with SCW in moso bamboo [47, 48]. The MYB gene family has specifically been reported to be involved in environmental responses [49]. In addition to rapid growth, the flowering pathway [50, 51] and sucrose synthase [52] have also been widely studied in bamboo. Recently, a chromosome-level de novo genome assembly of moso bamboo was provided, which, compared with the previous version, was obviously improved in terms of the assembly data and quality of the whole-genome sequencing assembly [43, 53]. The release of new bamboo genomic data allows us to perform genome-wide gene functional analyses in moso bamboo.

Here, we identified a total of 129 PhAP proteins that contain a conserved Asp domain from the moso bamboo genome. Phylogenetic analysis revealed that *PhAP* genes might have experienced gene expansion via segmental and tandem duplication. Gene structure and motifs indicated that the motifs of *PhAP*s were conserved, although the gene structure has changed throughout evolutionary history. Expression pattern analysis showed that *PhAP*s exhibited tissue-specific expression patterns, and several sets of *PhAP*s may play important roles during moso bamboo rapid growth. Our study provides a strong foundation for further research on the potential function of these proteins in bamboo development and an improved understanding of the AP gene family in fast-growing nontimber forest species.

## Results

### Genome-wide identification of AP genes from the moso bamboo genome

**Fig. 1:**
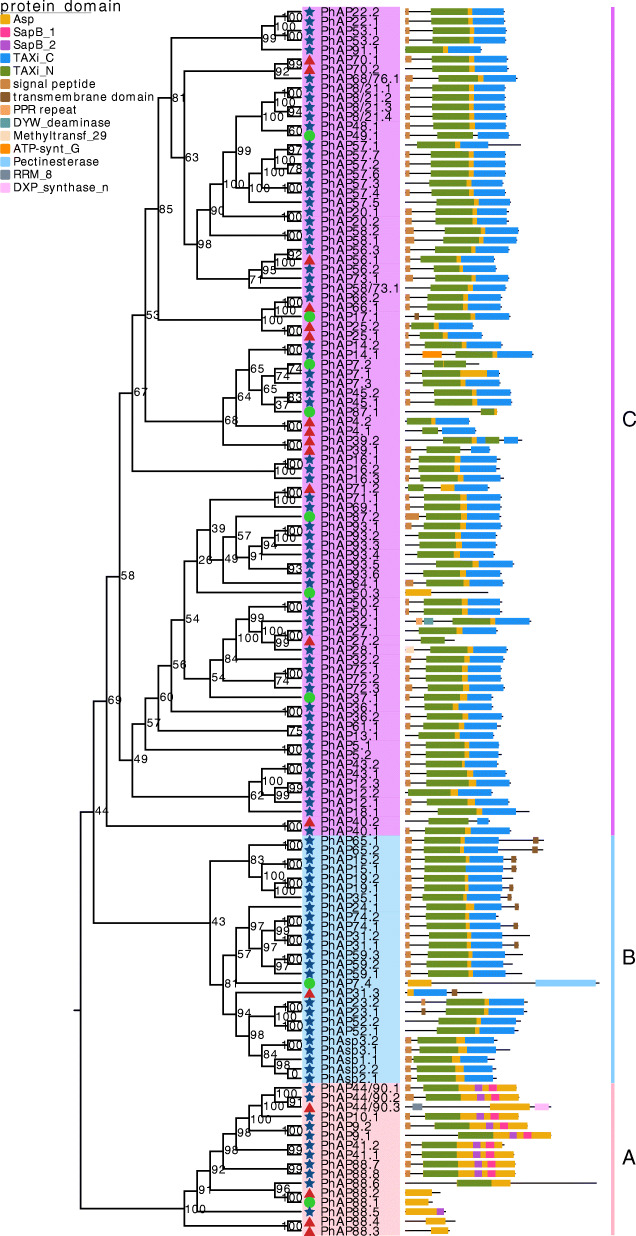
Phylogenetic relationships and protein domain diagram of moso bamboo aspartic proteinases. The left part shows the phylogenetic relationships of 129 APs from moso bamboo. Categories A, B And C are shaded in pink, blue and purple, respectively. The blue stars, red triangles and green circles represent APs containing 2, 1 and 0 catalytic sequences, respectively. Bootstrap are shown close to the branch nodes. The right part shows the protein domain, and the caption is shown in the upper left corner

After two rounds of moso bamboo genome searching via HMMER v3 (the details of which are in the materials and methods), a total of 129 Asp family proteins with a conserved Asp domain were analyzed via the NCBI-CDD and Pfam database (Fig. 1 and Table S1). Among these Asp proteins, 102 had two catalytic sequence motifs, Asp-Thr-Gly (DTG) and Asp-Ser-Gly (DSG), which are typical features of aspartic proteases; however, 18 proteins contained one catalytic motif, and nine proteins had no motif (Fig. 1 and Table S1). Moso bamboo Asp genes were named based on their relationships with homologous genes in rice and are listed in Table S1. Other information on the members of the Asp gene family, including their chromosomal localization, CDS, amino acid residue sequence, corresponding protein length, corresponding protein molecular weight, and corresponding protein isoelectric point, is also listed in Table S1.

Phylogenetic relationships among the 129 moso bamboo Asp proteins were determined using an IQ-TREE procedure [54]. The 129 moso bamboo Asp proteins fell into three distinct categories (pink, blue and purple clades) and were termed categories A, B and C, respectively (Fig. 1). From the predicted protein domain, we found that all *PhAP*s contained one Asp domain of variable length (Fig. 1). There were 16 moso bamboo category A *PhAP* members, eight of which contained signal peptides, and the Asp domain consisted of the Taxi_N and PSI domains (including SapB_1 and SapB_2) with two catalytic motifs (Fig. 1 and Table S1). However, there were no signal peptides or PSI domains and/or a lack or partial lack of catalytic motifs in the other eight category A *PhAP*s (Fig. 1 and Table S1). Categories B and C had 26 and 87 members, respectively, that contained the full-length Asp domain consisting of Taxi_N and Taxi_C, except for *PhAP7.4*, *PhAP31.3*, *PhAP7.2*, *PhAP87.1*, *PhAP4.1*, *PhAP50.3*, *PhAP27.2* and *PhAP40.2* (Fig. 1). Less than half of the category B *PhAP*s are nucellin-like APs containing catalytic sites (Fig. 1 and Table S1), which is similar to that which occurs rice [12]. Category C, composed of atypical aspartic proteases, was the largest category (Fig. 1). Most category B and C members contained a signal peptide, and it was notable that there were signal peptides and transmembrane domains located in the N- and C-termini, respectively, of nine category B AP proteins (Fig. 1).

### Phylogenetic analysis of APs from moso bamboo and rice

**Fig. 2:**
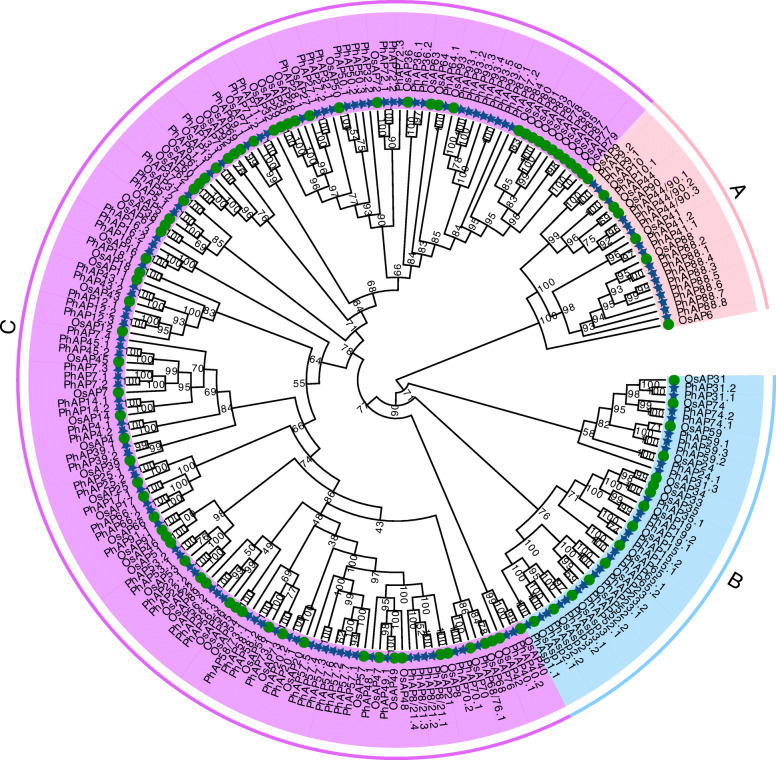
Phylogenetic tree of moso bamboo and rice aspartic proteinases. Categories A, B and C are shaded pink, blue and purple, respectively. The blue stars and green circles represent moso bamboo APs and rice APs, respectively. The bootstrap percentages are shown close to the branch nodes

To investigate the evolutionary relationship of the PhAP family, a phylogenetic tree was constructed using 129 PhAP and 92 OsAP full-length amino acid residue sequences (Table S1 and Table S2). Both PhAPs and OsAPs were classed into three categories, as previously reported in Arabidopsis [9], rice [12], grape [21] and poplar [22]. Category A contained 16 PhAPs together with seven OsAPs; these proteins could be classified into seven subclades based on their relationships with their rice homologous proteins (Fig. 2). There was at least one PhAP homolog in each subclade but no homolog of *OsAP6* (Fig. 2). The moso bamboo genome encoded eight PhAP88 genes and only one homolog in rice, which meant that AP88 underwent gene expansion in moso bamboo (Fig. 2). 25 *PhAP*s and 15 *OsAPs* were classified into category B, which could be further divided into 13 subclades (Fig. 2). Each subclade contained at least one PhAP homolog in moso bamboo (Fig. 2). Category C contained 87 *PhAP*s and 70 *OsAPs*. There was at least one PhAP homolog, and *PhAP57* and *PhAP93* exhibited evidence of gene expansion in moso bamboo (Fig. 2). There were no homologous genes of *OsAP77–87* in moso bamboo, indicating that the homologs in bamboo were lost during evolutionary history (Fig. 2). Altogether, these results showed that the *PhAP* gene family in moso bamboo underwent specific evolutionary events after the divergence of bamboo and rice.

### Chromosomal location and gene duplication events of *PhAP*s

**Fig. 3:**
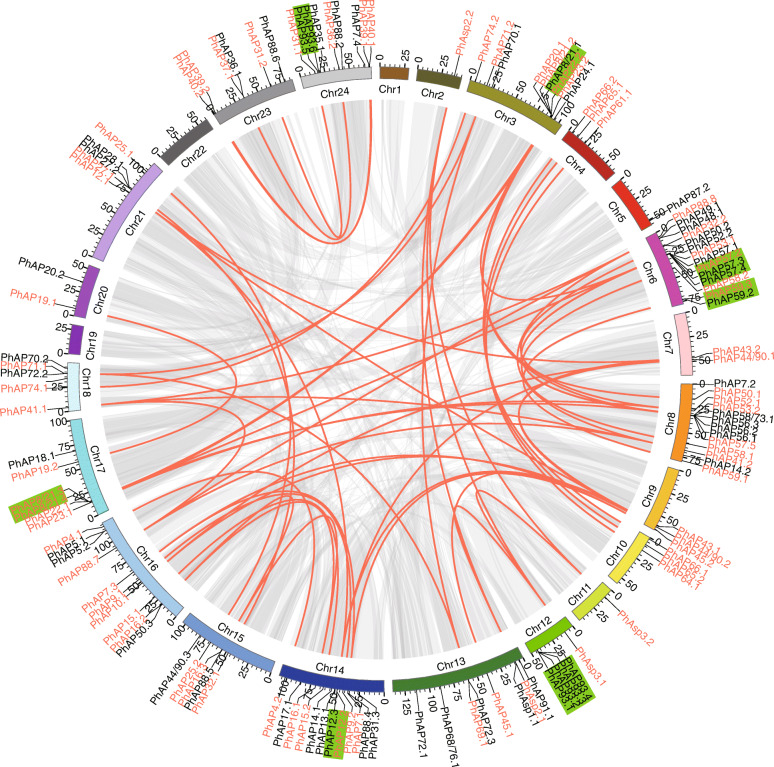
Chromosomal location and tandem duplicated genes among 124 *PhAP* genes. A total of 124 out of 129 *PhAP*s were mapped onto the chromosomes on the basis of their physical location. Chromosome numbers (Chr1- Chr24) are at the bottom of each chromosome. The gray lines indicate duplicated blocks, while the red lines indicate duplicated *PhAP* gene pairs. The genes listed in red font are segmentally duplicated, while tandemly duplicated genes are shaded in green

**Fig. 4:**
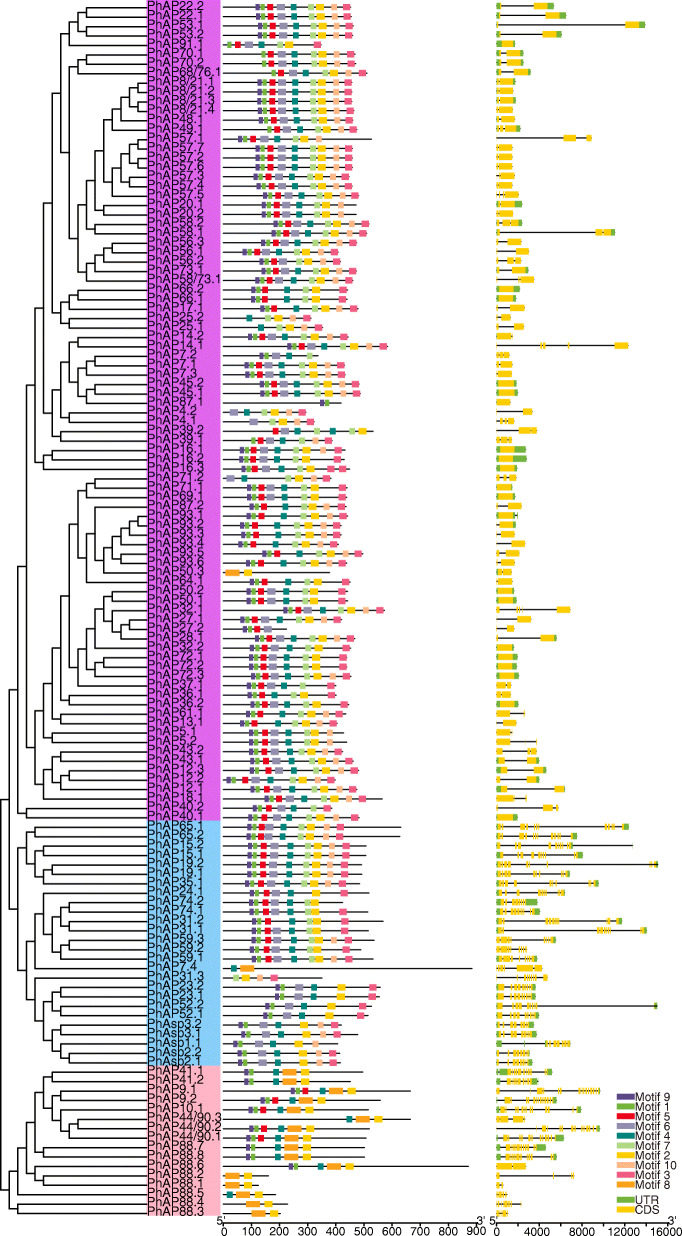
Phylogenetic tree of *PhAP*s as well as protein motifs and gene structure of corresponding *PhAP* genes from moso bamboo. The left panel shows the phylogenetic tree of *PhAP*s, as shown in Fig. 1. The middle panel shows the conserved protein motifs and their distribution. The boxes with different colors represent the conserved motifs listed in Fig. 5. The right panel shows the gene structure. The yellow boxes represent exons, the gray lines represent introns, and the green boxes indicate untranslated regions

We mapped the *PhAP*s onto chromosomes to examine the *PhAP* distribution on the moso bamboo chromosomes. Among the 129 *PhAP* genes, 124 were located on 21 out of 24 moso bamboo chromosomes, while the other five *PhAP*s were located on scaffolds (Fig. 3). Figure 3 shows that the chromosomal distribution of the *PhAP*s was nonrandom but was scattered and uneven. Fourteen *PhAP*s located on chromosome 6 contained the maximum number of *PhAP* genes; 13 *PhAP* genes were on chromosome 8; 12 *PhAP*s were on chromosome 14; and chromosomes 2, 5, and 11 had only one PhAP gene (Fig. 3). There was no *PhAP* gene located on chromosome 1, 19, or 22 (Fig. 4). Segmental and tandem duplications are considered to be the main reasons leading to gene family expansion in plants. As shown in Fig. 3, some *PhAP* genes (*PhAP8/21.1* and *PhAP8/21.2*; *PhAP57.2* and *PhAP57.3*; and *PhAP57.4*, *PhAP93.1* and *PhAP93.2* as well as *PhAP93.3* and *PhAP93.4*; *PhAP69.1* and *PhAP72.3*; *PhAP8/21.3* and *PhAP8/21.4*; and *PhAP93.5* and *PhAP93.6*) were adjacent to others and located sequentially in tandem on chromosomes 3, 6, 12, 14, 17 and 24, suggesting that these genes might have expanded via tandem duplication (Fig. 3). In addition to the tandem duplication, we also found that 73 *PhAP*s were located in segmental duplication blocks (Fig. 3). Furthermore, approximate divergence dates and the *K*a/*K*s ratio of duplication pairs were estimated (Table S3). The results showed that duplication events may have taken place from 63.41 million years ago (Mya) to 0.2 Mya. The Ka/Ks of the duplication pairs were < 1.0 except for *PhAP8/21.3* and *PhAP8/21.4*, *PhAP57.3* and *PhAP57.4*, and *PhAP93.1* and *PhAP93.2*, suggesting that most duplication pairs underwent purifying selection. These results indicated that most of the *PhAP* genes arose from tandem and segmental duplications, the processes of which play a very important role in the expansion of the *PhAP* gene family.

### Analysis of PhAP conserved motifs and gene structure

**Fig. 5:**
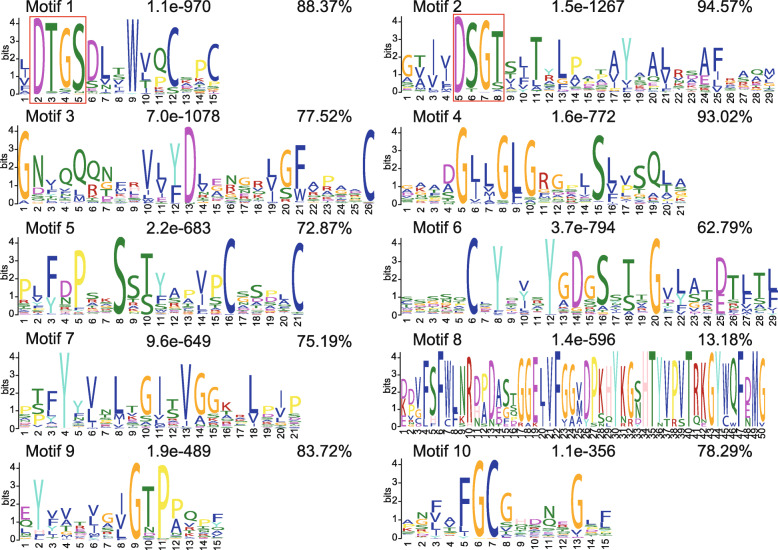
Conserved motif logos of *PhAP*s. The red boxes represent the catalytic sequence in motif 1 and motif 2. The number in the upper-middle of every motif is the *E*-value that represents the statistical significance of the motif. The percentage located in the upper right represents the enrichment percentage of each motif

The distribution of conserved protein motifs and gene structure are considered to play an important role in gene family evolution. First, we analyzed the conserved motifs of the PhAP proteins and their distribution by the MEME online tool. The conserved motifs and their distribution are shown in Fig. 4, while the corresponding logos are shown in Fig. 5. The conserved motif number in each of the *PhAP*s ranged from two to nine (Fig. 4). Motif 1 and motif 2 had the catalytic sequence motifs (Fig. 5) that were most conserved in the three categories and were present in nearly all members of *PhAP*s together with motif 4 and motif 9 (Fig. 4). Category A proteins had five or six conserved motifs, of which motif 8 was category A specific, with the exception of PhAP88.1–5 (Fig. 4). Motifs 3, 6, 7 and 10 were specific motifs of categories B and C (Fig. 4). Category B could be divided into two subclades based on the conserved motif distribution: the proteins in one clade had seven motifs, and the others had six motifs, except for PhAP7.4 and PhAP31.3 (Fig. 4). Like PhAP22s, the atypical category C members contained eight to nine motifs, but 18 members in category C had fewer than eight motifs because of the loss of some conserved motifs (Fig. 4). These results showed that the motifs were conserved in each category, although some PhAP members lost several motifs.

We further analyzed the exon/intron structure of the 129 *PhAP* genes (Fig. 4). *PhAP*s from different categories had distinct gene structures, which included exon/intron numbers, length and arrangement (Fig. 4). The gene structure of the category A genes was diverse (Fig. 4). *PhAP88.7* and *PhAP88.8* had 11 exons with similar exon numbers and distributions, whereas other homologs had two to eight exons of various lengths (Fig. 4); *PhAP88.1-PhAP88.8* were the closest homologs of *OsPhAP88* (Fig. 2). The other homologs in category A, such as *PhAP44/90s*, *PhAP9s* and *PhAP41s*, also had different gene structures (Fig. 4). The gene structure of members of category B was conserved because the close homologs had similar exon/intron structures, although there were different exon numbers and distributions between the two branches of category B (Fig. 4). The number of exons in category C was at most five, which was different from the numbers in *Populus* and grape [21, 22], and most of the closest homologs, such as *PhAP57s*, *− 14 s*, *− 7 s*, and *-4 s*, on exhibited different gene structures (Fig. 4). These results indicated that the gene structure of *PhAP*s changed throughout evolutionary history.

### *Cis*-element analysis of *PhAP* family genes

**Fig. 6:**
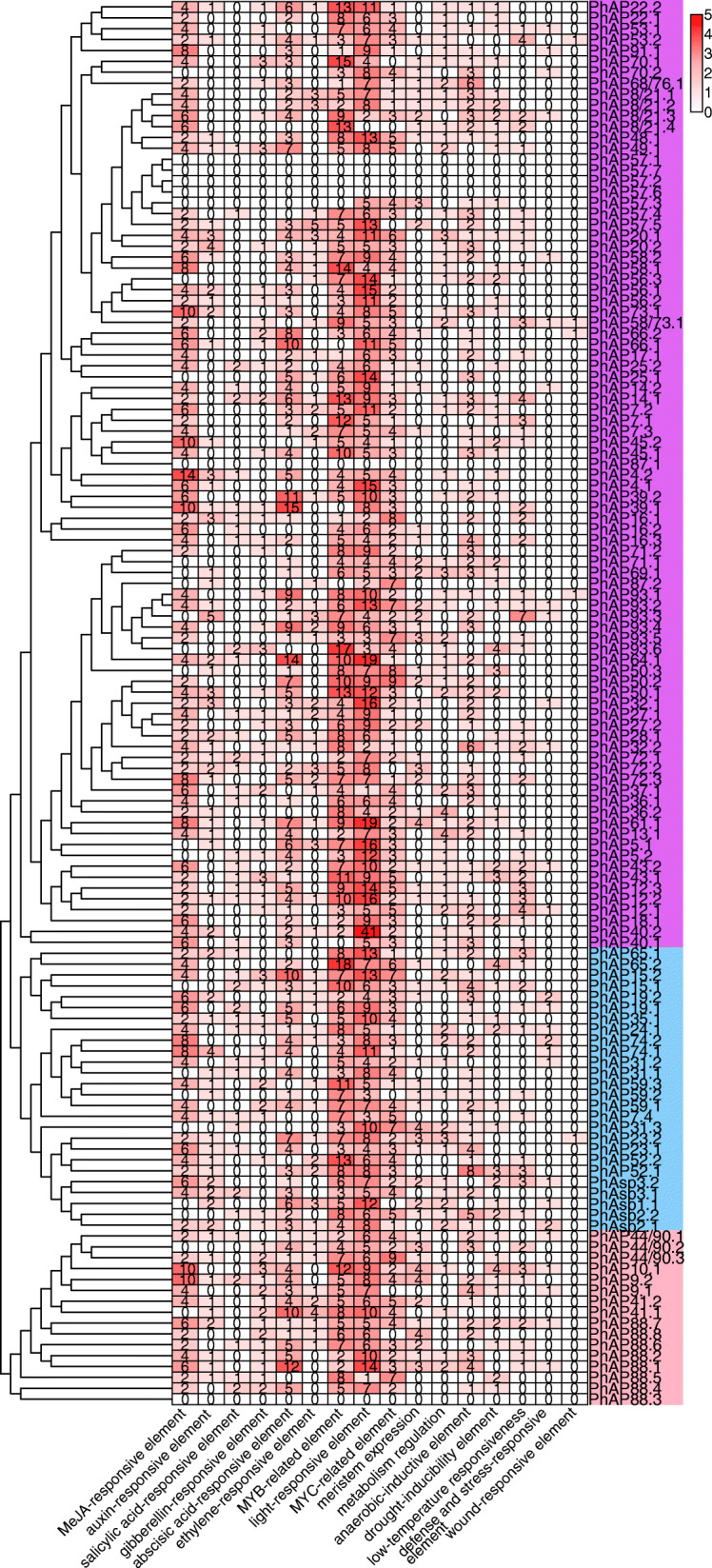
*Cis*-element analysis of the promoter region of *PhAP*s. The *cis*-elements located in the 1500 bp promoter sequence (upstream of the start codon) of all *PhAP*s were analyzed via the PlantCARE website. The number of corresponding *cis*-elements was used for the heatmap construction

*Cis*-elements are located in the promoter region of target genes and interact with transcription factors to trigger target gene expression. We herein filtered *cis*-elements of upstream sequences of the 129 *PhAP* genes (Fig. 6). It was very clear that the most abundant *cis*-elements located in the promoter region of *PhAP*s were MYB-related elements and light-responsive elements (Fig. 6). Nearly all *PhAP*s contained an average of seven MYB-related elements and nine light-responsive elements (Fig. 6). Forty-one light-responsive elements and 18 MYB-related elements existed in the promoter regions of *PhAP40.2* and *PhAP65.2*, respectively (Fig. 6). The second most abundant *cis*-elements were MeJA-responsive and abscisic acid-responsive elements, MYC-related elements and anaerobic-inductive elements (Fig. 6). In addition, 89 *PhAP*s had several anaerobic-inductive elements, 66 *PhAP*s contained one to four metabolic regulatory *cis*-elements, and 61 *PhAP*s contained relatively few *cis*-elements that were auxin-responsive elements and gibberellin-responsive elements (Fig. 6). A portion of the *PhAP* upstream regions contained a small number of meristem-related *cis*-elements (59 *PhAP*s), ethylene-responsive elements (57 *PhAP*s) and salicylic acid-responsive elements (42 *PhAP*s). Environmental stress-related *cis*-elements, such as drought-inducible elements, low-temperature-responsive elements, defense and stress-responsive elements, and wound-responsive elements, were also identified in some *PhAP* genes (Fig. 6). No *cis*-element was identified from *PhAP57.1*, − *57.7*, *− 57.2*, *− 57.6* or − *88.3* (Fig. 6). These results suggested that *PhAP*s may be widely involved in development and responses to environmental changes in moso bamboo.

Because SCW formation and PCD have been reported to occur during the moso bamboo rapid-growth stage, *PhAP* genes may be involved with key transcription factors that regulate SCW and PCD. We further scanned 10 *cis*-elements previously discovered to participate in SCW formation and PCD by MEME scanning [22, 55]. The results are shown in Figure S1. A total of 83% (107 out of 129 *PhAP*s) of *PhAP*s had at least one *cis*-element related to SCW formation and PCD. There were 64 and 50 *PhAP*s containing SMRE1 and SMRE3 *cis*-elements, respectively, and 27 *PhAP*s had ACIII and SMRE5 *cis*-elements. Seventy *PhAP*s had SMRE2 *cis*-elements. SMREs and ACIII are located in the promoter regions of secondary wall biosynthetic genes and are responsible for SCW [56]. Thirty-five *PhAP*s have ACII and TERE *cis*-elements. Similarly, 31 *PhAP*s had SNBE *cis*-elements. TERE and SNBE are critical *cis*-elements responsible for PCD and SCW during tracheary [37] and vessel [38] element formation, respectively. Sixteen *PhAP*s had s ACI and M46RE. The *cis*-element M46RE, which is recognized by MYB46, is also involved in SCW [57]. SCW- and PCD-related *cis*-elements were also found upstream of *PhAP57.1*, *− 57.2* and − *57.3* (Figure S1). To date, methyl jasmonate (MeJA)-, gibberellin (GA)-, ABA-, and ethylene-related as well as MYB transcription factors have been reported to be involved in PCD [58–60]. Hence, these results suggested that *PhAP*s may be potentially involved in SCW and PCD processes.

### Expression patterns of *PtAP* genes in moso bamboo tissues

**Fig. 7:**
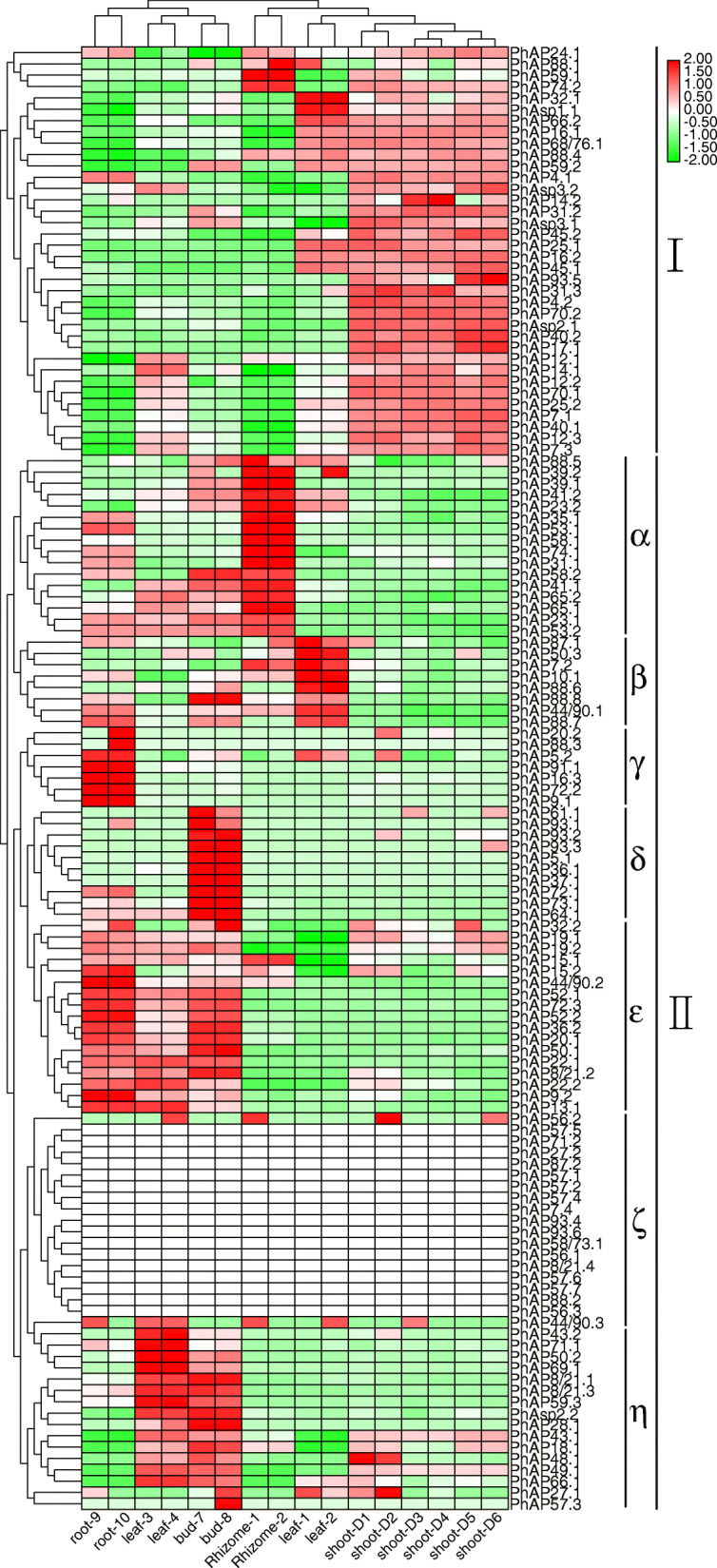
Expression patterns of *PhAP*s in different tissues of moso bamboo. The expression profiles of the 129 *PhAP*s is displayed by hierarchical clustering. The color bar in the upper-right corner indicates log2-based transcripts per million reads (TPM). Roots 9 and 10 represent 2 biological replicates of new roots with lateral roots, leaves 1 and 2 represent 2 biological replicates of leaf blades, leaves 3 and 4 represent 2 biological replicates of leaf sheaths, rhizomes 1 and 2 represent 2 replicates of rhizomes, and buds 1 and 2 represent 2 replicates of buds on rhizomes. Shoots D1 and D2, D3 and D4 and D5 and D6 represent 2 replicates of the top, middle and basal portions of the 6.7 m high shoot. The TPM value was listed in Table S4

To reveal the potential function of *PhAP*s, we analyzed the expression pattern of *PhAP*s in various tissues, including the roots, different portions of stems, leaf blades, leaf sheaths, buds, and rhizomes (Fig. 7), the data of which were obtained from publicly available RNA-seq data [43]. To check whether the RNA-seq data were reproducible, we randomly selected seven *PhAP*s and validated their expression patterns via RT-qPCR in different tissues of moso bamboo, including the top shoots, middle shoots, low shoots, leaves, sheaths, and roots. The results showed that the expression patterns of the seven *PhAP*s were consistent with the RNA-seq results (Figure S2). Figure 7 showed that the expression pattern of *PhAPs* were tissue preferences. The percentage of highly expressed *PhAP*s in each tissue is summarized in Figure S3A. On the basis of their expression patterns, the *PhAP*s were clustered into two main classes: class I and class II (Fig. 7). Almost all *PhAP*s showed transcript abundance in each tissue except class IIζ, which included *PhAP8/21.4*, − *57.1*, − *57.2*, − *57.4*, − *57.5*, − *57.6*, − *57.7*, − *56.1*, − *56.3*, − *58/73.1*, − *71.2*, − *87.2*, − *93.4*, − *93.6*, − *27.2*, − *7.4* and − *88.2*. Class I showed a high expression levels in the stems, although some members were highly expressed in the leaves or rhizomes (Fig. 7). In contrast, class II genes were nearly absent from the stems and showed various expression in the other above mentioned tissues (Fig. 7). Class II could be further divided into seven subclasses whose members exhibited preferential transcriptional abundance in the rhizomes (IIα); leaves (IIβ); roots (IIγ); buds (IIδ); roots, leaves and bud (IIε); and leaves and buds (IIη) (Fig. 7). No mRNA expression was detected from class IIζ members, which included 17 *PhAP*s from the abovementioned tissues (Fig. 7). Some *PhAP*s showed a highly specific expression pattern; for example, four *PhAP*s (*PhAP91.1*, − *16.3*, − *72.2* and − *9.1*) were mainly expressed in new roots with lateral roots, and four *PhAP*s (*PhAP71.1*, *− 50.3*, *− 43.2*, and *− 88.6*) were specifically expressed in the leaves (Fig. 7). Further, 12 *PhAP*s (*PhAP70.2*, *− 17.1*, − *7.1*, − *7.3*, − *45.2*, − *4.2*, − *93.5*, − *27.1*, − *40.1*, − *31.2*, − *31.3* and *2.1*) were expressed in only the shoots, and three *PhAP*s (*PhAP58.1*, *− 39.2* and − *88.1*) were expressed only in the rhizomes.

### Expression dynamics of *PhAP* genes in the rapid-growth stage of moso bamboo

**Fig. 8:**
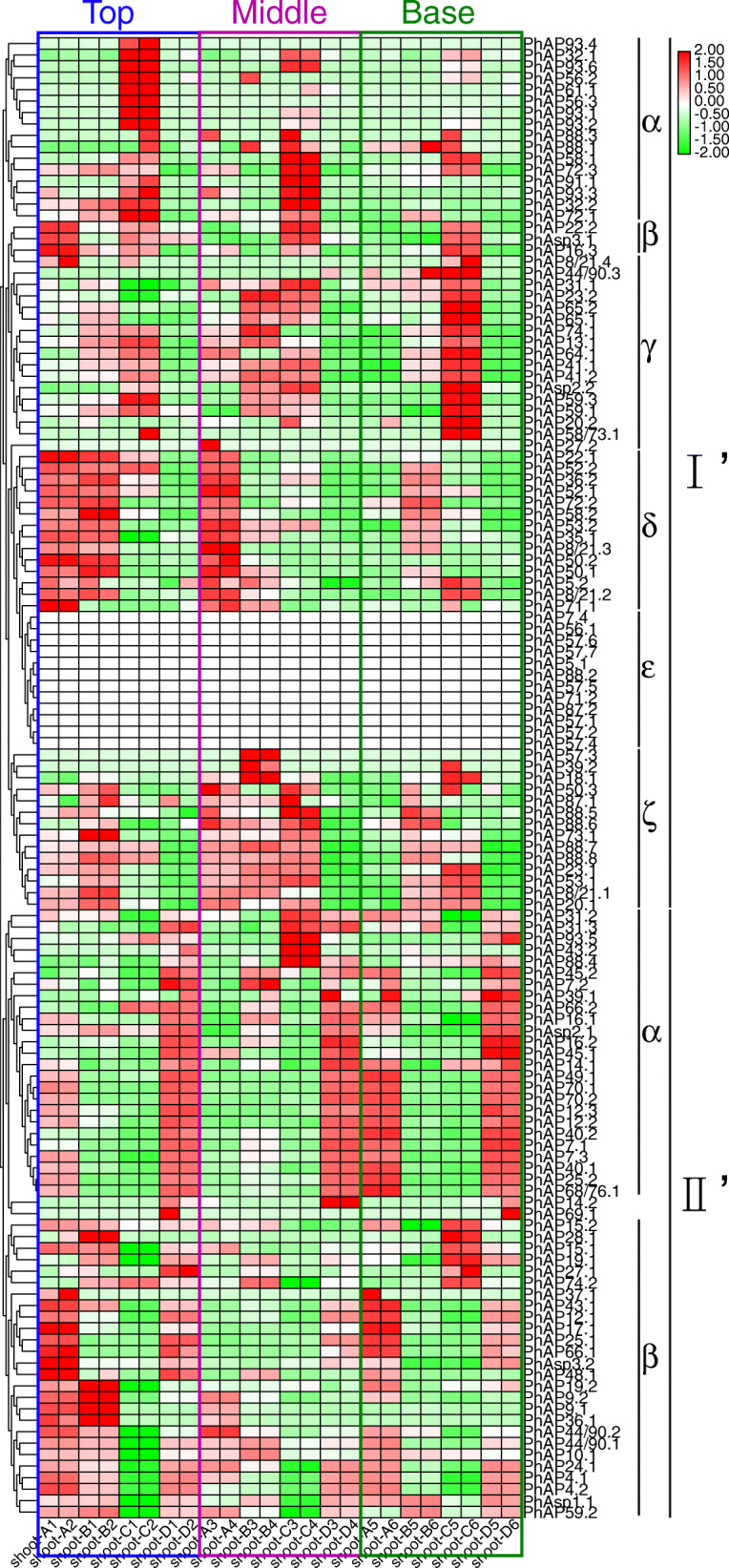
Expression heatmap of *PhAP*s in the top, middle and basal portions of rapidly growing shoots of moso bamboo. The expression profile of 129 *PhAP*s is displayed by hierarchical clustering. The color bar in the upper-right corner indicates log2-based TPM values. Shoots A, B, C, and D represent the basal portions of 0.2 m, 1.5 m, 3 m and 6.7 m high rapidly growing moso bamboo, respectively. The numerals 1 and 2, 3 and 4, and 5 and 6 represent 2 biological replications. The TPM was listed in Table S5

One of the most important features of moso bamboo is its rapid growth, which is mainly mediated by cell division and elongation [45]. Therefore, we further analyzed the expression patterns of the 129 *PhAP*s in the top, middle and basal portions of 0.2 m, 1.5 m, 3 m and 6.7 m tall moso bamboo, as previously reported [43]. Figure 8 shows that the expression patterns of the *PhAP*s also showed tissue specificity. The percentages of highly expressed genes in different parts of the shoots are summarized in Figure S3B. The *PhAP*s were divided into two main classes (class I′ and class II’) based on their expression abundance (Fig. 8). The transcript reads were detected, except for those of class I’ε (Fig. 8). All PhAP members of class I′ were nearly absent from the 6.7 m moso bamboo (Fig. 8). Class I’α members were highly expressed in the top portion of 3 m moso bamboo, and their transcript levels gradually decreased in the middle and basal portions (Fig. 8). In contrast, class I’γ transcripts accumulated in the basal portion of 3 m moso bamboo (Fig. 8), suggesting that the genes encoding those transcripts may be involved in cell elongation and SCW processes. Class I’δ transcripts preferentially accumulated in the top portion of the 0.2 m and 1.5 m moso bamboo shoots and in the middle portion of the 0.2 m moso bamboo shoots (Fig. 8); the genes encoding these transcripts may play critical roles in cell division. The mRNA level of class I’ζ members was slightly higher in the middle portion than in the other portions (Fig. 8). Compared with those of class I′, PhAP members of class II’ were expressed in 6.7 m moso bamboo (Fig. 8). For example, nearly all members from class II’α showed high mRNA abundance in the shoots of 6.7 m moso bamboo, although their expression was also detected in the basal portion of 0.2 m bamboo shoots (Fig. 8). Moreover, the members of class II’β had a high transcript level in the top portion of the 0.2 m and 1.5 m shoots. Taken together, these results suggested that specific *PhAP*s played a role in moso bamboo during different stages of the rapid-growth period.

**Fig. 9:**
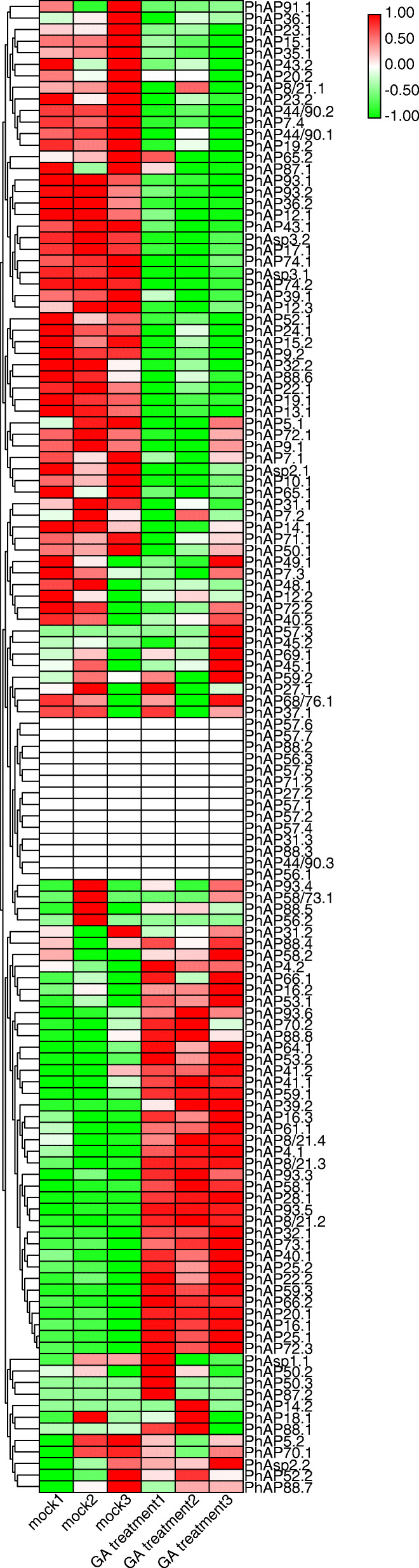
Expression heatmap of *PhAP*s in response to GA treatment. The expression profiles of the 129 *PhAP*s in response to GA (100 μM for 4 h) are displayed by hierarchical clustering. The color bar in the upper-right corner indicates log2-based expression values. The TPM was listed in Table S6

GA is considered to be one of the most important phytohormones involved in moso bamboo rapid growth, which includes cell expansion, SCW and PCD [44, 61]. APs have been reported to be involved in cell expansion and PCD processes and are associated with GA signaling [28, 62]. Therefore, using previously reported RNA-seq data, we analyzed whether *PhAP*s respond to GA treatment [56]. We further carried out RT-qPCR by randomly selecting seven genes to confirm the RNA-seq data. The leaves from two-month-old moso bamboo seedlings were treated with 100 μM GA3; the RT-qPCR results were consistent with the RNA-seq results (Figure S4). After GA treatment, 39.51% (32 *PhAP*s) and 43.21% (35 *PhAP*s) of *PhAP* expression levels were positively and negatively regulated, respectively, by GA, while the expression of 17.28% (14 *PhAP*s) of the *PhAP*s was not detected, and others exhibited poor reproducibility (Fig. 9 and Figure S3C). Almost all GA-responsive *PhAP* genes were involved in the rapid growth of moso bamboo (Figure S3). These results suggested that the *PhAP* genes may respond to GA signaling to regulate various aspects of the rapid growth of moso bamboo. Altogether, these results strongly suggested that most *PhAP*s play important roles in rapid growth.

## Discussion

As a class of proteolytic enzymes, APs are involved in protein processing and degradation. Increasing amounts of evidence have demonstrated that plant APs play critical functions in plant development and in response to biotic and abiotic stresses. With the completion of whole-genome sequencing of a large number of plant species, members of AP gene families have been identified from additional plant species, such as *Arabidopsis*, poplar, rice and grape. However, little information is known about the *AP* gene family in moso bamboo, which is one of the fastest growing plant species on Earth. In this study, we identified a total of 129 moso bamboo *PhAP*s that have conserved protein domains and gene structures and that are evolutionary related to members of the *OsAP* gene family. Expression pattern analysis also indicated that these *PhAP*s may be involved in the development of different organs and in rapid growth-related processes in moso bamboo.

APs were widely distributed across plant species, and we identified 129 *PhAP* genes from the moso bamboo genome, the number of which was greater than that of rice (96 *OsAP*s) [12], poplar (67 *PtAP*s) [22], *Arabidopsis* (69 *AtAP*s) [9], and grape (50 *VqAP*s) [21]. The 129 *PhAP* members could be classified into three categories: category A (16 *PhAP*s), category B (25 members), and category C (88 members) (Figs. 1, 2 and Table S1). Category A was the smallest category, and category C revealed gene expansion had occurred for its members, which was similar to findings in *Arabidopsis*, rice, poplar and grape [9, 12, 21, 22]. Perhaps the formation of these three categories occurred before the monocotyledon and dicotyledon divergence. When they were compared with those from *Arabidopsis*, grape, poplar and rice, the *PhAP*s in moso bamboo revealed gene expansion had occurred for members in each category, especially categories A and B, which were approximately two times larger than they were in rice (Fig. 2). It has been reported that moso bamboo has experienced whole-genome duplication, which led to more gene duplication events [53]. We found that 56.6% (73 *PhAP*s) of *PhAP*s were located in duplication blocks (Fig. 3), the percentage of which was greater than that in rice (24.7%, *23 OsAP*s) [12] and grape (32.0%, 16 *VvAP*s) [21] but less than that in poplar (62.6%, *42 PtAP*s) [22]. In addition, we also identified tandem duplication events in the *PhAP* family, such as *PhAP8/21 s*, *PhAP57* and *PhAP93* (Fig. 3). Category C of *PhAP*s also underwent gene expansion, as determined for *PhAP57* and *PhAP93*, but some homologs of OsAPs, such as *OsAP77–87*, were lost during the evolutionary process (Fig. 3). These results suggested that, owing to the different development and growth features, compared with those in rice, APs in moso bamboo underwent a distinct evolutionary pathway. For example, moso bamboo exhibits rapid growth characteristics in the early growth stage [43, 45, 59].

The protein domain of *PhAP*s was conserved during the evolutionary process. For example, all members contain a conserved full-length Asp domain with a catalytic sequence (Fig. 1); few *PhAP*s had no catalytic sequence, similar to those in rice and poplar [12, 22]. Signal peptides and transmembrane domains were also present in homologous genes of moso bamboo, such as those in rice, *Arabidopsis*, poplar and grape [9, 12, 21, 22] (Fig. 1). Motifs 1 and 2 contained the catalytic sequence, and motifs 4 and 9 were conserved and present in nearly all members of the three categories (Figs. 4 and 5), which is similar to that described in poplar [22]. Members of each category shared the conserved motif (Fig. 4), and the members of each category contained a specific motif, for example, motif 8, which was category A specific, and motifs 3, 6, 7 and 10, which were category B and C specific (Fig. 4). These results suggested that motifs of the PhAP family are diverse. The gene structures of *PhAP*s from the same category were more conserved than those from different categories (Fig. 4). The gene structure of the members of category B was most conserved, while that of category A and category C was less conserved and was similar to the gene structure of the corresponding members of category A and category C in grape and poplar but different from those in rice [12, 21, 22]. These results suggested that *PhAP*s may have originated from an ancient DNA sequence and experienced expansion with variation. The domain varied among the three categories, although the Asp domain and catalytic sequence were conserved, suggesting that there may be functional divergence.

Gene expression is specifically controlled in space and time, and the precise regulation by developmental and environmental signals are essential for gene function. AP genes are reported to be widely expressed in plants [9, 12, 21, 22]. *PhAP*s had high transcript abundance in nearly all tissues, including the roots, leaves, buds, shoots and rhizomes, although no transcripts were detected for a few *PhAP*s (Fig. 7). Some *PhAP*s also exhibited tissue-specific expression patterns; for example, four *PhAP*s and three *PhAP*s were specifically highly expressed in the roots and rhizomes, respectively (Fig. 7). These specific expression patterns were similar to those in other plant species. For example, three AP genes were not detected in the tested tissues, while other APs were generally expressed in root leaves, stems, flowers, fruits and tendrils with various abundances in grape [21]. In poplar, AP genes exhibited preferential expression patterns in the mature and young leaves, roots, female and male catkins and xylem [22]. In addition, *cis*-elements reported to respond to environmental change, phytohormones, PCD and SCW were located in the promoter region of APs in moso bamboo (Fig. 6 and Fig. 1). These results indicated that AP genes may function in various tissues in moso bamboo and are potentially regulated by various developmental and environmental cues. The mechanism of *PhAP* tissue-specific expression patterns and the contribution of various factors is an interesting question worthy of further study.

Rapid growth of moso bamboo was observed shortly after the young shoots emerged from the ground [43]. GA is a critical phytohormone involved in rapid growth, and the spatial and temporal distribution of GA is strictly regulated [44, 61]. The top portion of young bamboo shoots contain GA at a concentration higher than that in the basal portion [45]. Pith cavity formation accompanied by PCD and cell division are the main biological events in the top portion during the moso bamboo rapid-growth stage [44, 45]. We found that 59 *PhAP*s were expressed in the top region of the 0.2 m moso bamboo shoots (Fig. 8). Among them, *PhAP25s*, which is the ortholog of *OsAP25* in rice and is triggered by PCD in plants [35], was highly expressed at the top and was expressed at low levels in the middle or basal part (Fig. 8). Notably, *PhAP25s* was activated by GA treatment (Fig. 9). These results suggest that *PhAP25s* may be regulated by GA and may play a role in pith cavity formation in the early stage of rapid bamboo growth. In the middle and basal portions, cell elongation coupled with cell wall thickening is fundamental [45]. PhAP members from class I’α, class I’γ and class II’α were highly expressed in the middle and basal portions (Fig. 8). Among them, the mRNA abundance of *PhAP65.1*, *PhAP65.2* and *PhAP19.1* peaked in the basal portion of the 3 m moso bamboo shoots (Fig. 8). In rice, *OsAP65* is involved in pollen germination and pollen tube growth [32]. The homologs of *PhAP19.1* in *Arabidopsis* were *A36* and *A39*, which encode two aspartic proteases that are preferentially transcribed in pollen and that affect pollen tubes and tube cell wall deposition [18]. These results suggested that *PhAP*s highly expressed in the middle and basal portions may play functions in cell elongation and cell wall component formation. It is interesting that not all APs highly expressed in the top part were activated by GA or that those highly expressed in the basal part were repressed by GA. One reason is that GA is not the unique regulatory factor of *PhAP*s; these genes may be regulated by other developmental and environmental signaling pathways. How *PhAP* expression is precisely controlled requires further investigation.

Because the shell of bamboo covers the stem of young shoots, which presented a yellow-like etiolation status [43, 44], the rapid growth of moso bamboo was similar to skotomorphogenesis, including protein degradation processes [63]. We found that light-responsive elements were located in nearly all *PhAP* upstream promoter regions (Fig. 6). It was reported that the A1 expression level in *Arabidopsis* is upregulated by light [64]. We also found that nearly all *PhAP* expression levels were dynamic at different stages (Fig. 8). The phytohormone GA has been reported to be involved in skotomorphogenesis, and the GA concentration reached its maximum in the basal portion of moso bamboo [45, 65]. Most *PhAP*s also positively or negatively respond to GA treatment (Fig. 9), although a small amount of GA-responsive *cis*-elements were identified in the promoter region of the *PhAP*s (Fig. 6). Most *PhAP*s may not be directly regulated by GA during the moso bamboo rapid-growth stage. Expression of APs regulated by GA was previously reported in *Arabidopsis*; for example, *ASPG1*, which is critical for seed germination, is activated by GA treatment [28]. This indicated that *PhAP*s involved in rapid growth are activated to participate cell division or elongation during the different stages. Altogether, these results strongly suggest that *PhAP*s play critical roles in rapid growth of moso bamboo by integrating developmental and environmental signaling.

## Conclusion

Aspartic proteases are important proteolytic enzymes that function in plant development and in response to environmental changes. In this study, a total of 129 *PhAP*s were identified from moso bamboo, a fast-growing plant species. The PhAP family in moso bamboo underwent gene expansion via segmental and tandem duplications that were distinct from those in rice. Notably, several sets of PhAP genes showed dynamic transcript abundance during the moso bamboo rapid-growth stage, suggesting that *PhAP*s may play critical roles in moso bamboo rapid growth by mediating environmental and developmental signaling.

## Methods

### Genome resources

Chromosome-level reference genomes of moso bamboo (*Phyllostachys edulis*) and whole rice (*Oryza sativa*) plants were downloaded from the GigaDB Database [43] (http://gigadb.org/dataset/100498) and Phytozome database (https://genome.jgi.doe.gov/portal/pages/dynamicOrganismDownload.jsf?organism=Osativa), respectively.

### Identification of members of the asp gene family

To identify moso bamboo Asp genes, predicted proteins from the moso bamboo genomic database were searched by HMMER v3 [66] using the hidden Markov model (HMM) file that corresponded to the Asp domain (PF00026) downloaded from the Pfam database (http://pfam.xfam.org/) as a query [67]. The obtained protein sequences with an expected value (E) < 1E-20 and containing the Asp domain were aligned by ClustalW [68] and used to construct a moso bamboo-specific HMM file via hmmbuild from HMMER v3. The new moso bamboo-specific Asp HMM was used as a query against the predicted proteins of moso bamboo. All peptide sequences with E < 1E-2 and containing the Asp domain identified by the Pfam database [67] and NCBI-CDD tools [69] were selected as Asp genes (Table S1). *OsAP* genes were reported previously [12], we confirmed them in Phytozome database and listed in Table S2.

### Multiple sequence alignment and phylogenetic and domain analyses

129 *PhAP*s and 92 OsAPs amino acid residues were aligned by MEGA 7.0. The alignment files were uploaded to the IQ-TREE web server [54] (http://iqtree.cibiv.univie.ac.at/) for phylogenetic tree construction, with the default parameters. The protein domains were identified by searching the Pfam database. EvolView [70–72] (https://www.evolgenius.info/evolview/#login) and Dendscope 3 [73, 74] were used for phylogenetic tree visualization and annotations.

### Protein motif and gene structure analyses

The conserved motifs of the PhAP genes were identified by the MEME Suite web server [55] (http://meme-suite.org/). The number of motifs was set to 10, and all other parameters were the default ones. The gene structure and conserved domain were visualized via TBtools [75].

### Chromosomal distribution and gene duplication

The length of the chromosomes and the locations of the PhAP genes in the moso bamboo genome were used to map genes onto the chromosomes through MapChart [76]. Nucleotide sequences with alignment ratios and similarity ratios greater than 70% and with distances between genes on the same chromosome of less than 100 kb were selected as tandem duplications. Moreover, genes located in duplication regions and nucleotide sequences with alignment ratios greater than 75% were selected as resulting from segmental duplications [77–80]. The *K*a/*K*s values were calculated by TBtools [75]. The formula T = *K*s/2r was used to estimate the divergence time of PhAP gene pairs in moso bamboo. The r for moso bamboo and rice was 6.5 × 10^− 9^ years [81]. A diagram of collinearity analysis of genes and chromosomes was constructed using Circos [82].

### *Cis*-element analysis

The 1500 bp upstream DNA sequence of the 5′-UTR of the *PhAP* genes was selected as the promoter sequence. The promoter sequences were uploaded to the PlantCARE database (http://bioinformatics.psb.ugent.be/webtools/plantcare/html/) to scan for *cis*-elements. The *cis*-elements from the PlantCARE database were subsequently screened manually. The identification and location of previously reported PCD- and SCW-related *cis*-elements (SNBE, TERE, M46RE, ACI, ACII, ACIII, SMRE1, SMRE2, SMRE3 and SMRE5) were scanned via the MEME Suite web server [55].

### Expression analysis of *PhAP* genes

The raw RNA-seq data for new roots with lateral roots; blades; leaf sheaths; buds on rhizomes; rhizomes; and top, middle and basal portions of bamboo shoots of different heights (0.2 m, 1.5 m, 3 m and 6.7 m) were retrieved from the GigaDB dataset [43]. The raw RNA-seq data for GA treatment were retrieved from NCBI Sequence Read Archive (SRA) under the accession number GSE104596. Quantification of transcript expression was carried out by Salmon [83], and TPM were obtained for further analysis.

### RT-qPCR analysis

Moso bamboo tissue materials (top, middle and basal portions of the shoots, leaf blades, sheaths and roots) were harvested from 8 m-high bamboo growing in a bamboo forest at Fujian Agriculture and Forestry University (FAFU). Two-month-old seedlings were treated with 100 μM GA3 for 4 h [61]. The control groups were treated with the same concentration of DMSO instead of 100 μM GA3 for 4 h. Samples (three independent replications) were collected, immediately frozen in liquid nitrogen and stored at − 80 °C.

Total RNA of the samples was extracted using an RNAprep Pure Plant Plus Kit (Tiangen, China). Total RNA was then reverse transcribed using a PrimeScript RT Reagent Kit together with gDNA Eraser (Takara, China). RT-qPCR was performed via GoTaq qPCR Master Mix (Promega, USA). *PhUBQ* was used as an internal control gene [61]. All the primers used in this study are listed in Table S7. The reaction mixture consisted of 10 μl of 2× GoTaq qPCR Master Mix, 0.4 μl of each gene-specific primer, 1 μl of cDNA and 8.2 μl of nuclease-free water. The reaction conditions were as follows: 95 °C for 2 min followed by 40 cycles of 95 °C for 15 s and 60 °C for 20 s. The relative gene expression levels were calculated by the comparative ΔCt method (2^-ΔCt^). Three biological replications were assessed per sample.

## Supplementary Information

**Additional file 1: Table S1.** The information of AP family genes in *Phyllostachys edulis.*

**Additional file 2: Table S2.** The information of AP family genes in *Oryza sativa*.

**Additional file 3: Table S3.** Estimated divergence time of AP gene pairs in *P. edulis*.

**Additional file 4: Figure S1.** Analysis of PCD- and SCW-related *cis*-elements in the PhAP promoters.

**Additional file 5: Figure S2.** Expression level of seven selected *PhAP*s in different tissues of moso bamboo.

**Additional file 6: Figure S3.** Percentage of *PhAPs* highly expressed in different tissues, in response to GA treatment and at different developmental periods of shoots.

**Additional file 7: Figure S4.** Expression level of seven selected *PhAP*s after GA treatment.

**Additional file 8: Table S4.** The expression data of *PhAPs* in different tissues.

**Additional file 9: Table S5.** The expression data of *PhAPs* at different developmental periods of moso bamboo shoot.

**Additional file 10: Table S6.** The expression data of *PhAPs* under GA treatment.

**Additional file 11: Table S7.** RT-qPCR primers used for *PhAP* genes.

## Data Availability

All data supporting the conclusions of this article are provided within the article and its additional files. The genomics sequences data of moso bamboo and rice are available in the GigaDB Database (http://gigadb.org/dataset/100498) and Phytozome database (https://genome.jgi.doe.gov/portal/pages/dynamicOrganismDownload.jsf?organism=Osativa), respectively. The public RNA-seq data are available on GigaDB Database (http://gigadb.org/dataset/100498) and NCBI GEO under the accession number GSE104596.

## Abbreviations

AP: Aspartic protease
APs: Aspartic proteases
PCD: Programmed cell death
ASPG1: ASPARTIC PROTEASE IN GUARD CELL 1
CDR1: CONSTITUTIVE DISEASE RESISTANCE 1
ASPR1: ATYPICAL ASPARTIC PROTEASE IN ROOT 1
EAT1: ETERNAL TAPETUM 1
PCS1: PROMOTION OF CELL SURVIVAL
SCW: Secondary cell wall
TPM: Transcripts per million reads
