# Supplementary material for: Genome-wide identification, evolution and expression analysis of the aspartic protease gene family during rapid growth of moso bamboo (Phyllostachys edulis) shoots
Source: BMC Genomics. 2021 Jan 10;22:45. doi: 10.1186/s12864-020-07290-7 (PMC7798191; doi:10.1186/s12864-020-07290-7)
Supplement: Supplementary file 4 — Additional file 4: Figure S1. Analysis of PCD- and SCW-related cis-elements in the PhAP promoters. [file 12864_2020_7290_MOESM4_ESM.docx]

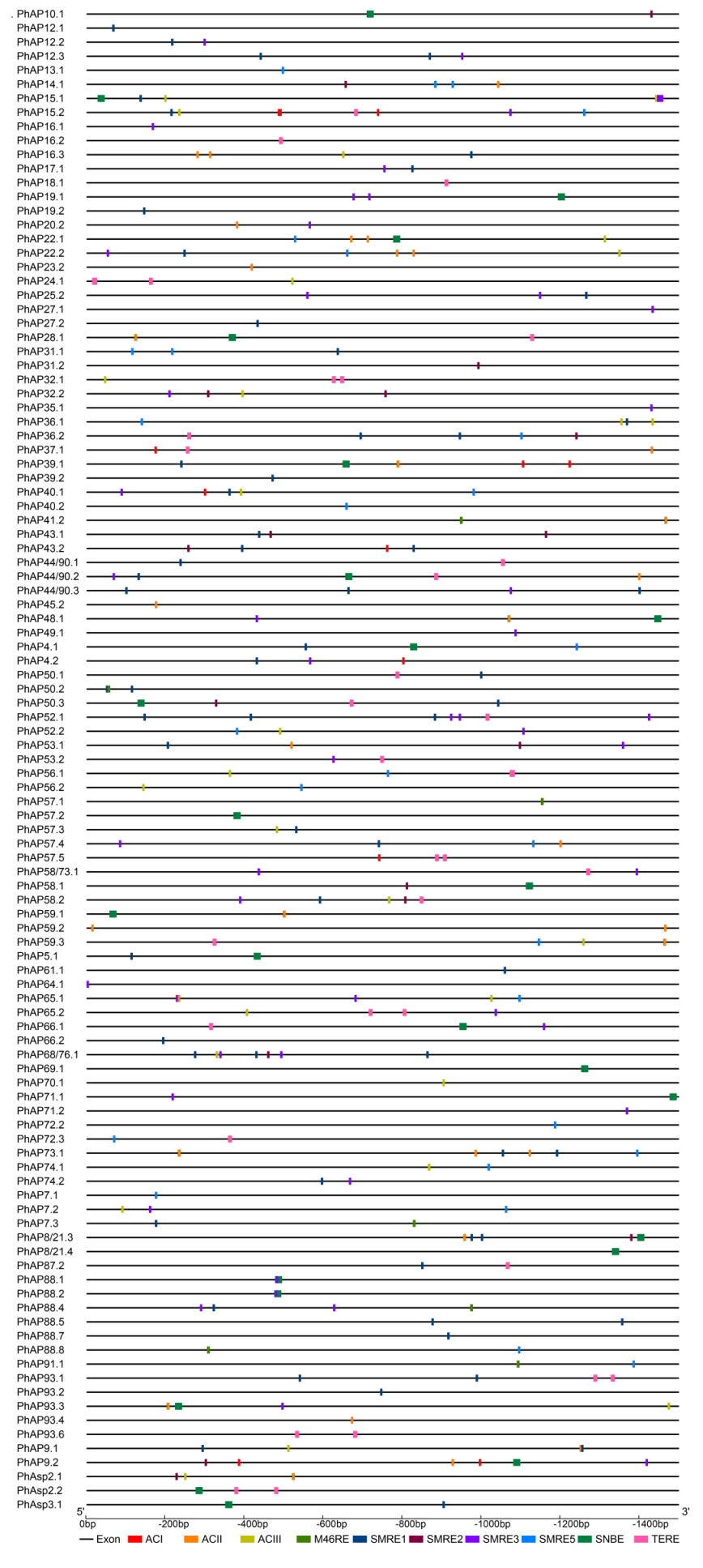


**Figure S1.** Analysis of PCD- and SCW-related *cis*-elements in the *PhAP* promoters. The 1,500 bp promoter sequences of all *PhAPs* were scanned by the 10 reported PCD and SCW *cis*-element via MEME suite. Each *cis*-element is indicated by a color box.
