# Supplementary material for: Genome-wide identification, evolution and expression analysis of the aspartic protease gene family during rapid growth of moso bamboo (Phyllostachys edulis) shoots
Source: BMC Genomics. 2021 Jan 10;22:45. doi: 10.1186/s12864-020-07290-7 (PMC7798191; doi:10.1186/s12864-020-07290-7)
Supplement: Supplementary file 5 — Additional file 5: Figure S2. Expression level of seven selected PhAPs in different tissues of moso bamboo. [file 12864_2020_7290_MOESM5_ESM.docx]

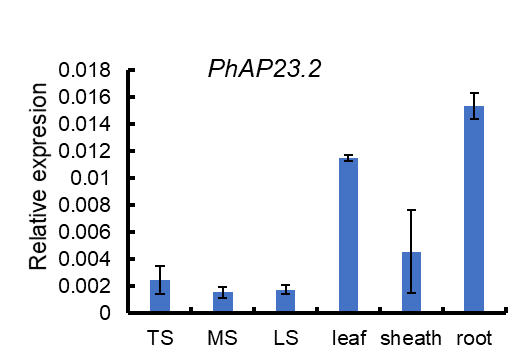


***

**


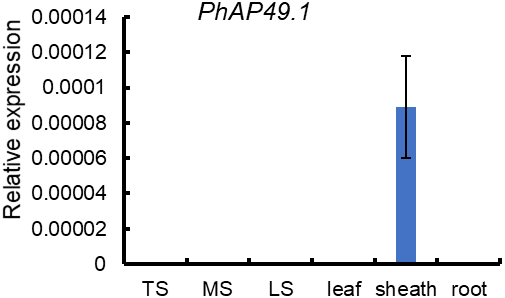


**


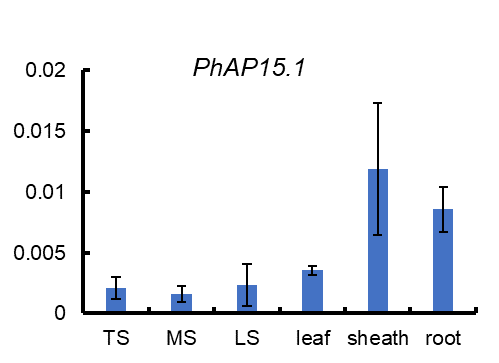


**

**


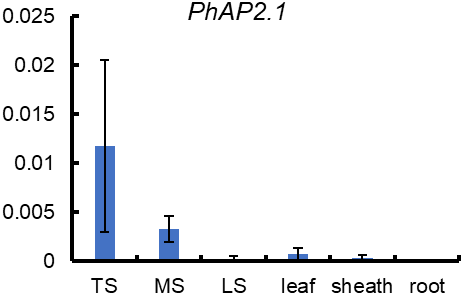


**

**


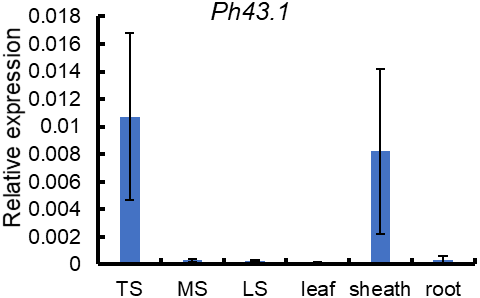


**

***


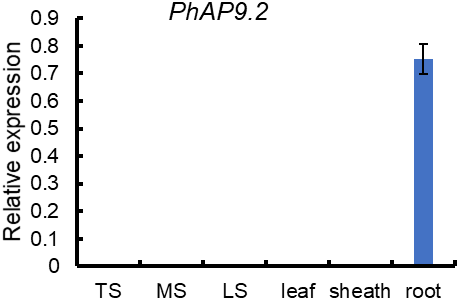


**


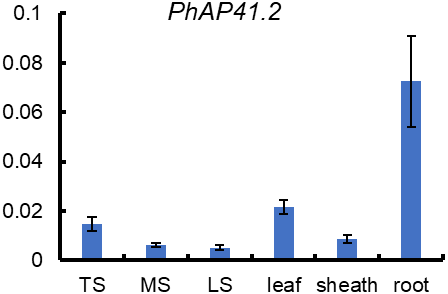


**

**

**Figure S2.** Expression level of seven selected *PhAPs* in different tissues of moso bamboo. *PhUBQ* was used as internal control. TS, MS and LS represent shoot-D1 and D2, shoot-D3 and D4, shoot-D5 and D6, leaf represent leaf-1 and 2, sheath represent leaf-3 and 4, root represent new root with lateral roots in Figure 7. **P<0.01 in three-biological repeats *t* test comparing with others samples.
