# Supplementary material for: Genome-wide identification, evolution and expression analysis of the aspartic protease gene family during rapid growth of moso bamboo (Phyllostachys edulis) shoots
Source: BMC Genomics. 2021 Jan 10;22:45. doi: 10.1186/s12864-020-07290-7 (PMC7798191; doi:10.1186/s12864-020-07290-7)
Supplement: Supplementary file 6 — Additional file 6: Figure S3. Percentage of PhAPs highly expressed in different tissues, in response to GA treatment and at different developmental periods of shoots. [file 12864_2020_7290_MOESM6_ESM.docx]

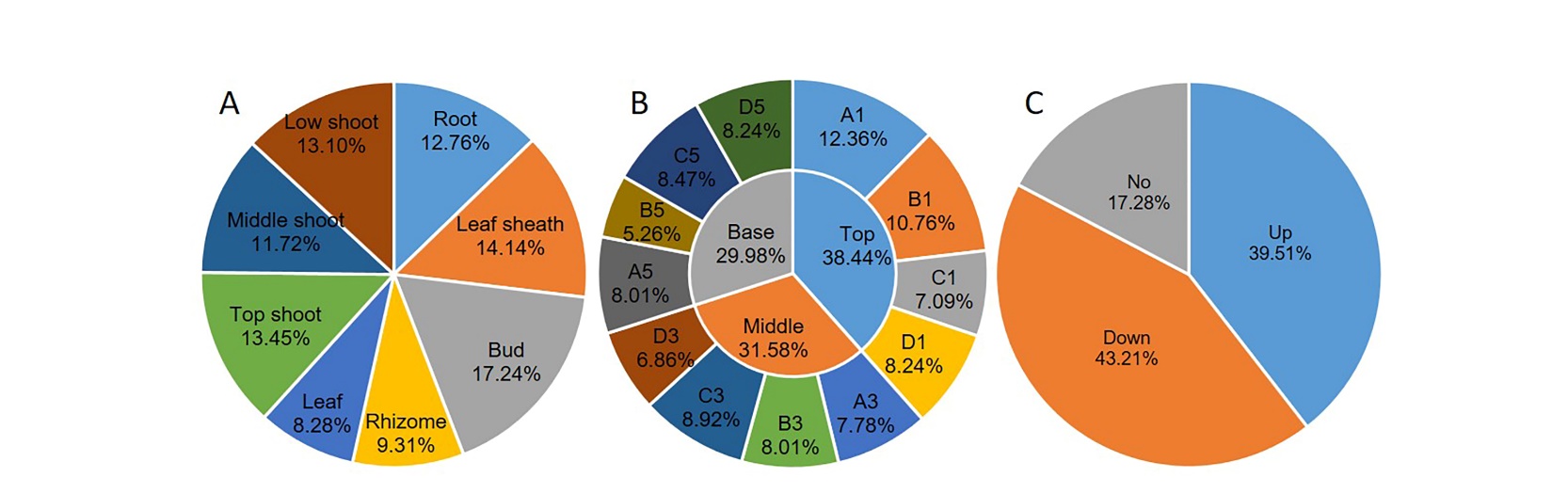


**Figure S3.** Percentage of *PhAPs* highly expressed in different tissues, in response to GA treatment and at different developmental periods of shoots. A. Percentage of *PhAPs* expressed in different tissue. Root, Leaf sheath, Bud, Rhizome, Leaf, Top shoot, Middle shoot, Basal shoot represent root-9, root-10, leaf-3, leaf-4, bud-7, bud-8, rhizome-1, rhizome-2, shoot-D1, shoot-D2, shoot-D3, shoot-D4, shoot-D5, and shoot-D6 in Figure 7, respectively. B. Percentage of *PhAPs* expressed in fast growth of shoot. Basal, Middle, Top represent three portions of shoot. A, B, C, D represent the same portion of 0.2 m, 1.5 m, 3 m and 6.7 m high moso bamboo. C. Percentage of *PhAPs* response to GA3 treatment (100 μM). Up and Down represent up and down regulated after GA treatment, respectively. No represents no expression change after GA treatment.
