# Supplementary material for: Genome-wide identification, evolution and expression analysis of the aspartic protease gene family during rapid growth of moso bamboo (Phyllostachys edulis) shoots
Source: BMC Genomics. 2021 Jan 10;22:45. doi: 10.1186/s12864-020-07290-7 (PMC7798191; doi:10.1186/s12864-020-07290-7)
Supplement: Supplementary file 7 — Additional file 7: Figure S4. Expression level of seven selected PhAPs after GA treatment. [file 12864_2020_7290_MOESM7_ESM.docx]

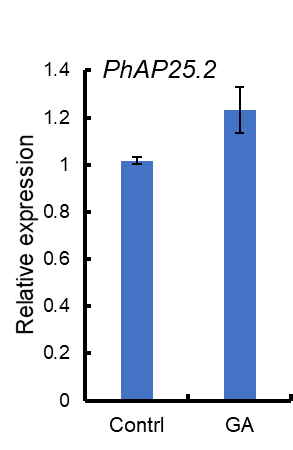


**


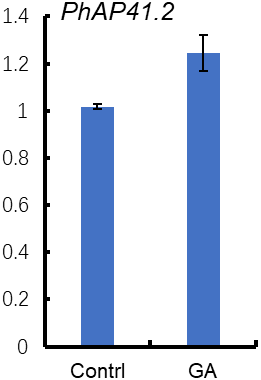


**


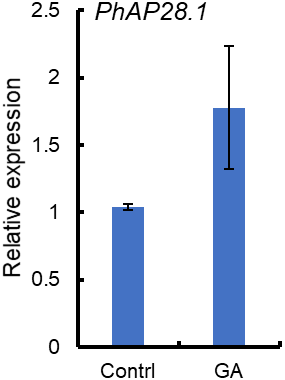


**


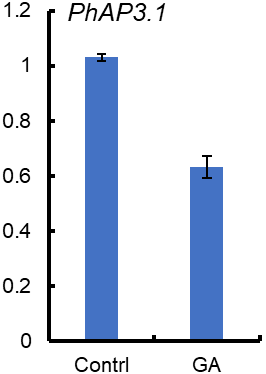


**


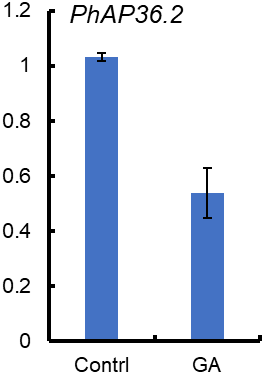


**

*


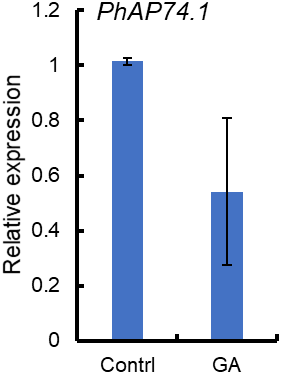


**


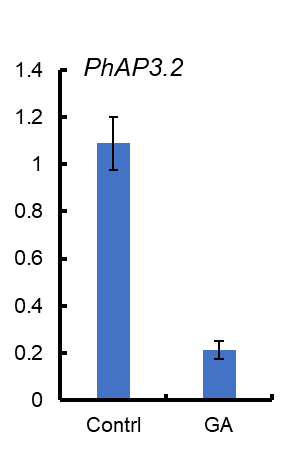


**

**Figure S4**. Expression level of seven selected *PhAPs* after GA treatment. *PhUBQ* was used as internal control. **P<0.01 in three-sample *t* test comparing with control.
